# Supplementary figures and images for: Whole-genome resequencing reveals collagen-related genes in Kele pigs
Source: PLoS One. 2024 Dec 31;19(12):e0311417. doi: 10.1371/journal.pone.0311417 (PMC11687657; doi:10.1371/journal.pone.0311417)

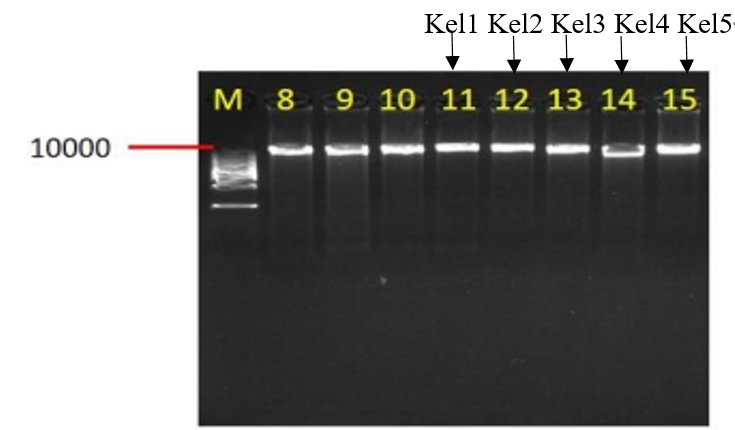

Supplement: S1 File — (ZIP) [file pone.0311417.s001.zip › Supporting Information/S1_Fig.tiff]

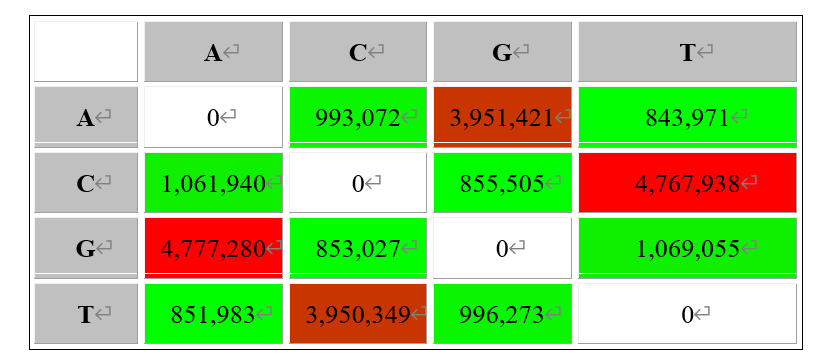

Supplement: S1 File — (ZIP) [file pone.0311417.s001.zip › Supporting Information/S2_Fig.tiff]

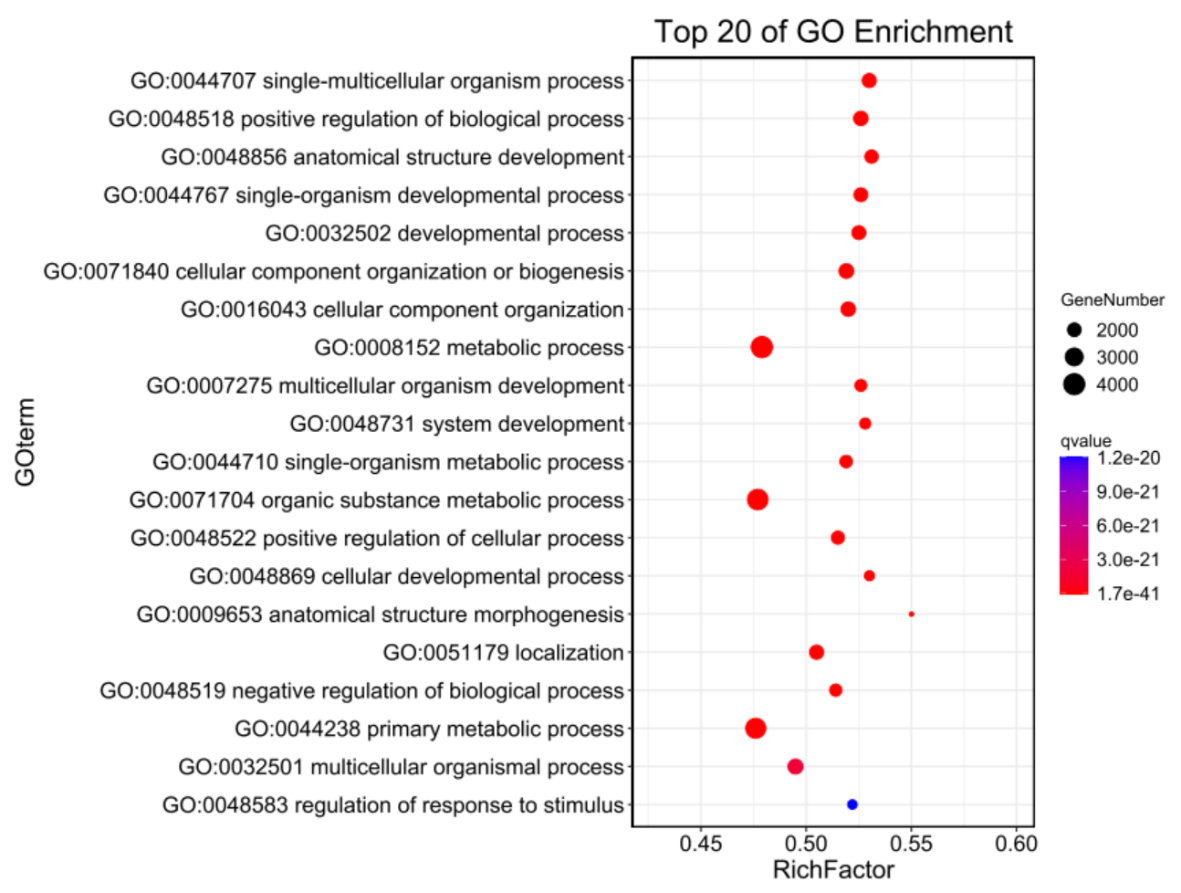

Supplement: S1 File — (ZIP) [file pone.0311417.s001.zip › Supporting Information/S3_Fig.tiff]

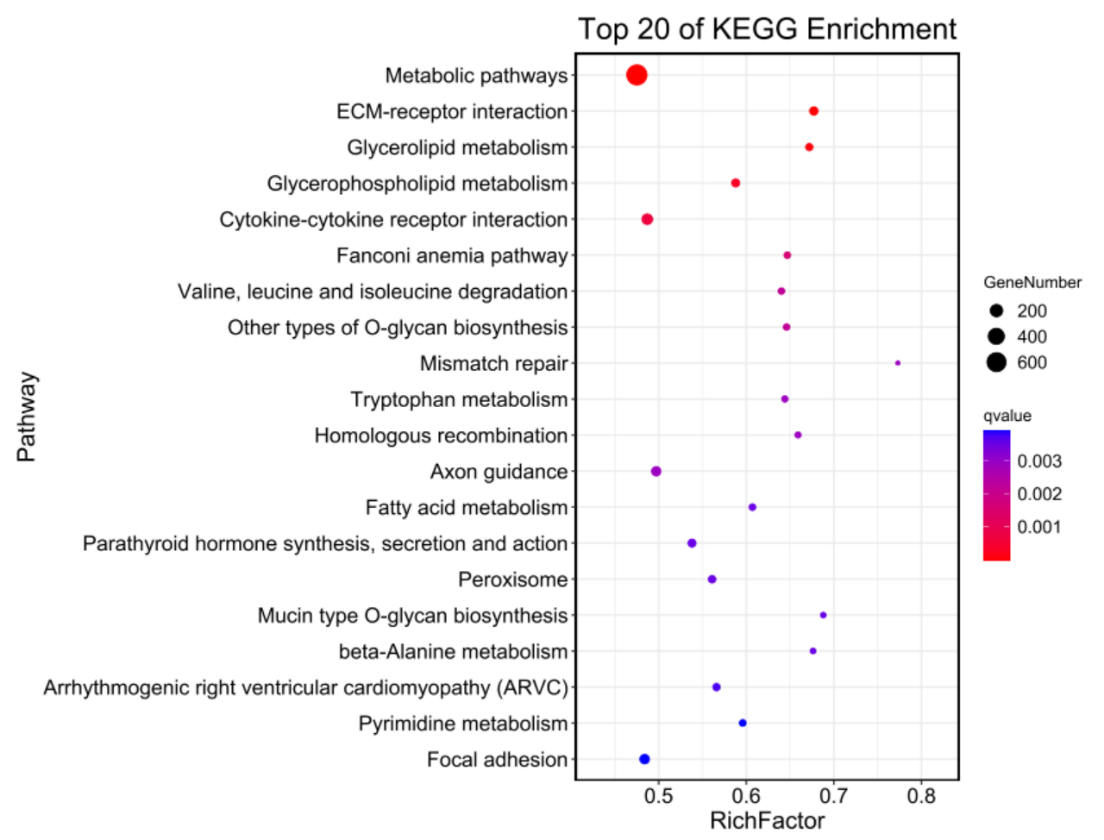

Supplement: S1 File — (ZIP) [file pone.0311417.s001.zip › Supporting Information/S4_Fig.tiff]

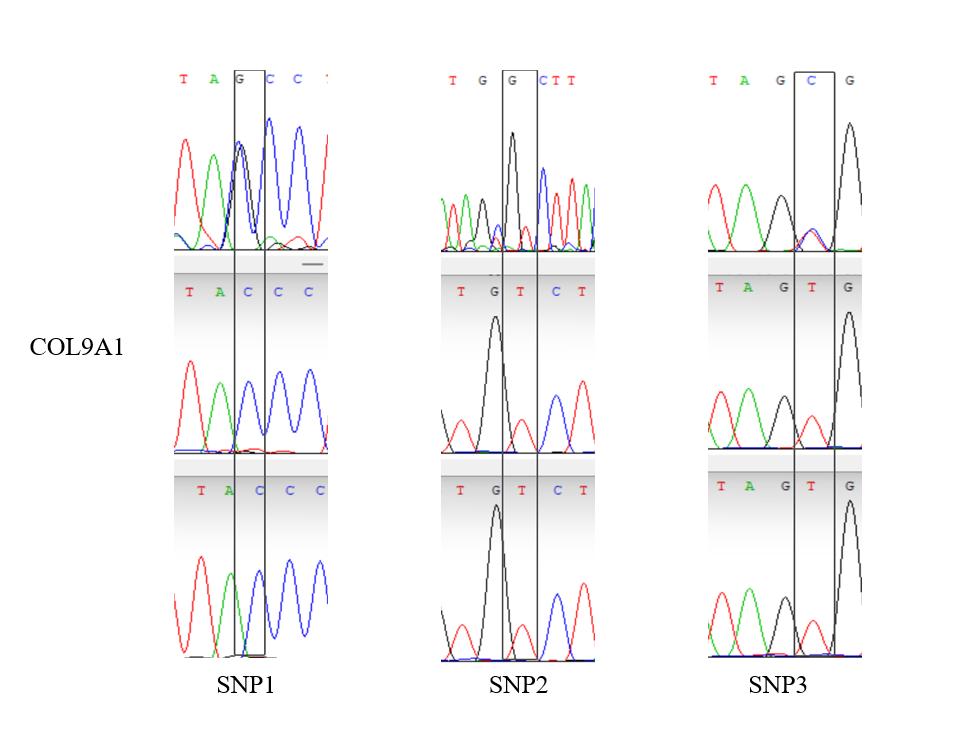

Supplement: S1 File — (ZIP) [file pone.0311417.s001.zip › Supporting Information/S5_Fig.tiff]

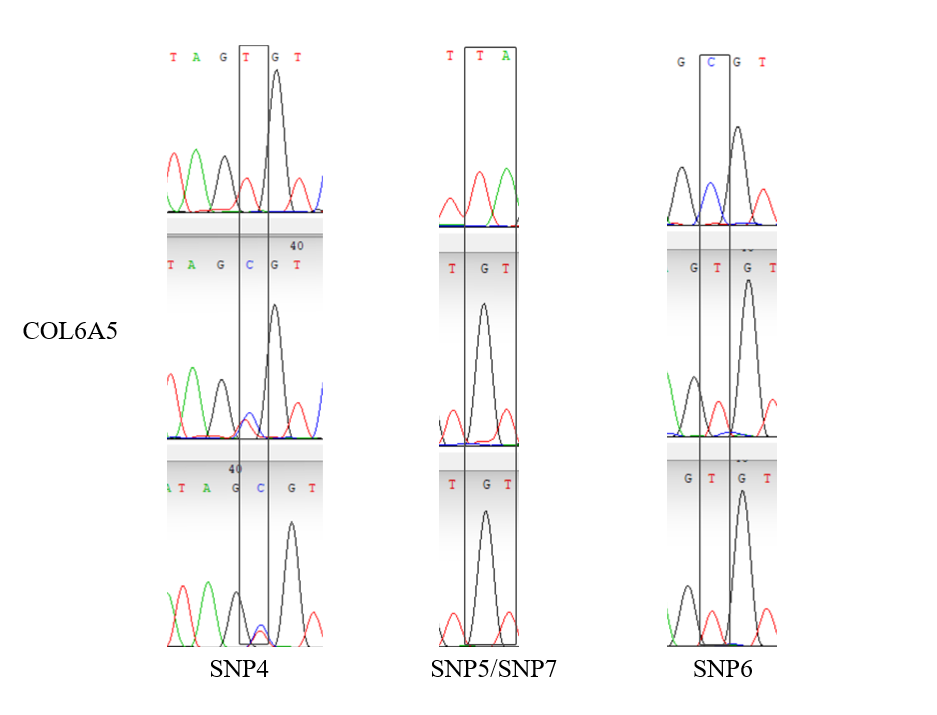

Supplement: S1 File — (ZIP) [file pone.0311417.s001.zip › Supporting Information/S6_Fig.tiff]

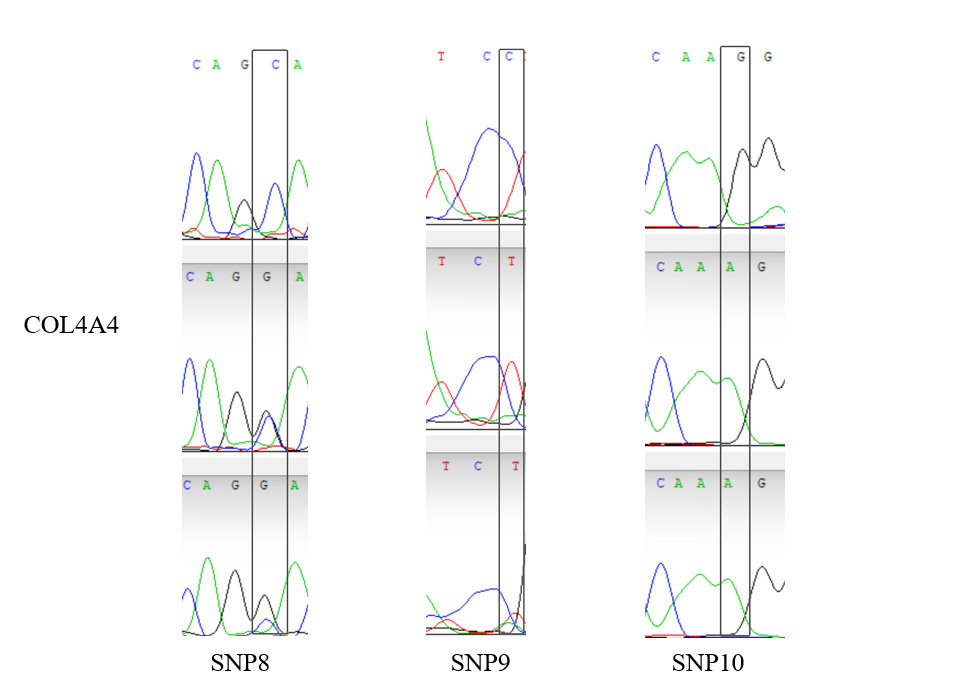

Supplement: S1 File — (ZIP) [file pone.0311417.s001.zip › Supporting Information/S7_Fig.tiff]

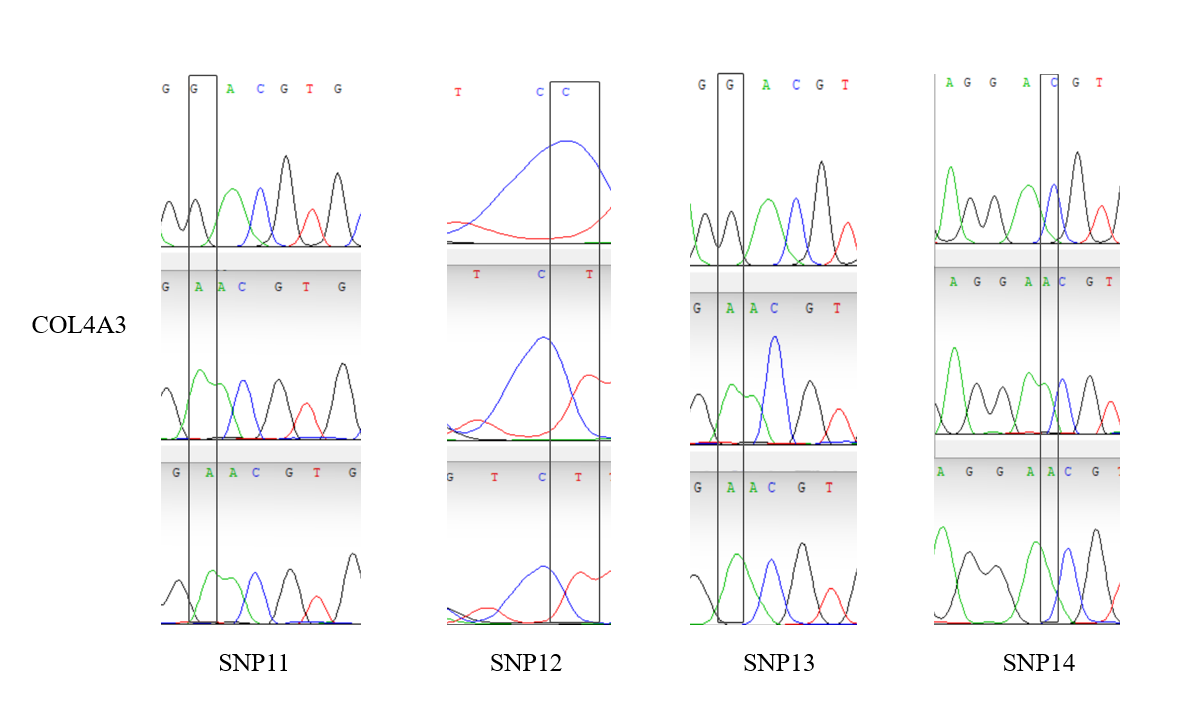

Supplement: S1 File — (ZIP) [file pone.0311417.s001.zip › Supporting Information/S8_Fig.tiff]

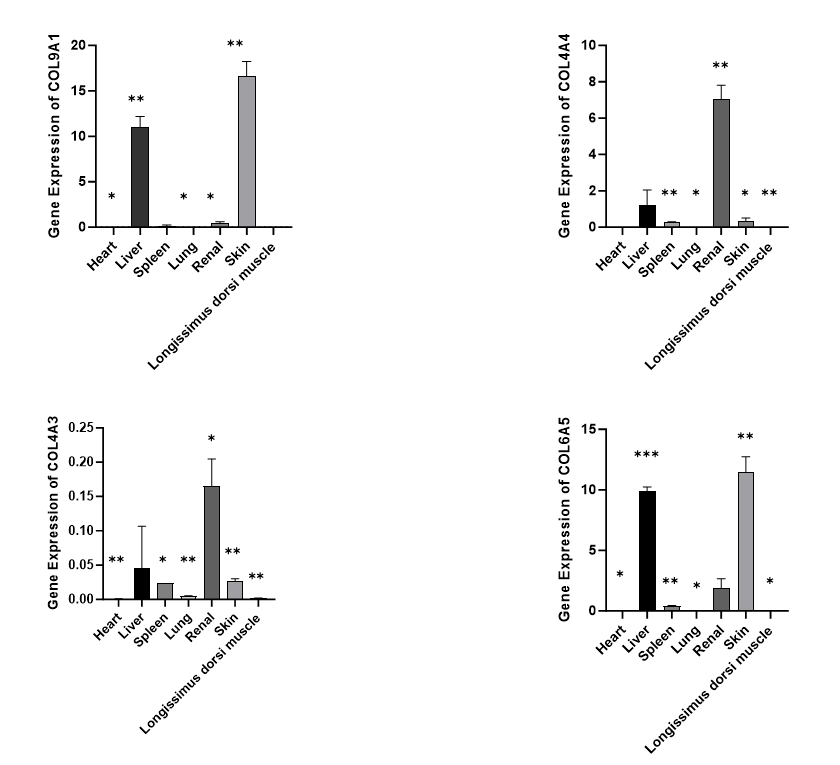

Supplement: S1 File — (ZIP) [file pone.0311417.s001.zip › Supporting Information/S9_Fig.tiff]
