# Supplementary material for: Whole-genome resequencing reveals collagen-related genes in Kele pigs
Source: PLoS One. 2024 Dec 31;19(12):e0311417. doi: 10.1371/journal.pone.0311417 (PMC11687657; doi:10.1371/journal.pone.0311417)
Supplement: Attachment — Submitted filename: Comments.docx [file pone.0311417.s002.docx]

Recommendation: Major and Minor revisions

Comments:

The topic is very interesting and the experiments were carefully performed by authors. This article highlights several genes that could be considered potential candidates for encoding collagen proteins in future research.

Thus, I recommend this article for publication in PLOS ONE after the following major and minor points are addressed by the authors.

Major Revision:

1. Authors' results indicated that the expression levels of COL9A1, COL6A5, COL4A4 and COL4A3 were investigated using RT-qPCR but are there any other supporting experiments done to confirm this overexpression of these genes?

Minor Revision:

1. What is the exact concentration of genomic DNA used in agarose gel electrophoresis?
2. In this manuscript some DNA samples A260/A280 is near to 2, but generally A260/A280 should be in the range of 1.8–1.9.
3. Except the fine meat quality and excellent flavour, are there any significant reasons to choose Kele pig as a model?
4. The conclusion is not impressive in its current form. Conclusion section of the manuscript requires further development to strengthen its impact and clarity.

5. It is recommended to include references to the recently published manuscripts on collagen-related genes to ensure the manuscript reflects the latest advancements in the field.
6. Authors should ensure that the manuscript is free from typos and grammatical errors.
