# Supplementary material for: Whole-genome resequencing reveals collagen-related genes in Kele pigs
Source: PLoS One. 2024 Dec 31;19(12):e0311417. doi: 10.1371/journal.pone.0311417 (PMC11687657; doi:10.1371/journal.pone.0311417)
Supplement: Attachment — Submitted filename: Comments.docx [file pone.0311417.s003.docx]

1. I did not find it relevant to utilize SNP/INDEL extraction from whole genome resequencing data to reveal the collagen related genes in Kele pigs from different tissues as it should have done using RNA-seq data as you are validating the change in level of expression using RT-PCR. Objective and conclusion of your study is failed to explain utilization of SNP/INDEL from whole genome resequencing data in place of RNA-seq data, which is the standard approach to access the expression of genes. Relevant explanation of it should be added and how its better to use SNP/INDEL extraction from whole genome resequencing data.
2. Title, abstract and headings should be improved.
3. There is no methodology provided for genomic localization (eg. Exon etc.) of SNP/INDELs. Further, no methodology is provided to get impact of SNP/INDELs and for gene annotation of the SNP/INDELs as directly mentioned the KEGG analysis which is only possible after extraction of candidate genes of SNP/INDELs.
