# Supplementary material for: Whole-genome resequencing reveals collagen-related genes in Kele pigs
Source: PLoS One. 2024 Dec 31;19(12):e0311417. doi: 10.1371/journal.pone.0311417 (PMC11687657; doi:10.1371/journal.pone.0311417)
Supplement: Attachment — Submitted filename: Response to Reviewers.docx [file pone.0311417.s004.docx]

Many thanks to all reviewers for their valuable comments and professional comments on this study and on us. We sincerely thank the editors and all reviewers for their feedback, which we will use to improve the quality of the manuscript.

Based on the main and secondary questions raised by reviewer 1, we have answered them as follows:

Major Revision:

1. Authors' results indicated that the expression levels of *COL9A1*, *COL6A5*, *COL4A4* and *COL4A3* were investigated using RT-qPCR but are there any other supporting experiments done to confirm this overexpression of these genes?

Based on whole genome resequencing, our study explored the genes related to collagen-protein traits of *Kele pigs*, and found the expression levels of four genes, *COL9A1*, *COL6A5*, *COL4A4* and *COL4A3*, respectively in different tissues of *Kele pigs*, such as heart, liver, spleen, lung, kidney, skin and longissimus dorsi muscle. It provided the basis for further research on how to regulate collagen-protein traits of *Kele pig.* In addition, our study also supplemented the experimental part: sanger sequencing was performed to verify the SNP variation sites of the four genes *COL9A1*, *COL6A5*, *COL4A4* and *COL4A3* selected by whole genome resequencing, and the results were consistent with expectations, laying a foundation for the later molecular application regulation. Here are the results of sanger's sequencing validation:

**Fig 5 Peak map of SNPs locus with variation in Sanger sequencing of *COL9A1* gene**

**Fig 6 Peak map of SNPs locus with variation in Sanger sequencing of *COL6A5* gene**

**Fig 7 Peak map of SNPs locus with variation in Sanger sequencing of *COL4A4* gene**

**Fig 8 Peak map of SNPs locus with variation in Sanger sequencing of *COL4A3* gene**

Major Revision:

1. What is the exact concentration of genomic DNA used in agarose gel electrophoresis?

The exact concentrations of genomic DNA used in agarose gel electrophoresis are tabulated below, and the exact concentrations of genomic DNA used in agarose gel electrophoresis have been supplemented in revised manuscripts and manuscripts.

| **Serial number** | | **Sample name** | | **Concentration**  **(ug/ml)** | |
| --- | --- | --- | --- | --- | --- |
| 1 | *Kel1* | | 55.6 | |  |
| 2 | *Kel2* | | 378 | |  |
| 3 | *Kel3* | | 442 | |  |
| 4 | *Kel4* | | 812 | |  |
| 5 | *Kel5* | | 580 | |  |

2. In this manuscript some DNA samples A260/A280 is near to 2, but generally A260/A280 should be in the range of 1.8–1.9.

As for the sample DNA extraction experiment, the value of A260/A280 was indeed high. In the subsequent pcr gel electrophoresis experiment, we found that the gel electrophoresis results showed that the extracted genomic DNA strips of *Kele pig* were single and clear with high concentration. All the DNA of the 5 samples met the requirements for the sequencing of the database, so the follow-up experiment was continued. We are very grateful to the review experts for correcting our mistakes and shortcomings. In the later study and work, we will treat each experiment more rigorously.

3. Except the fine meat quality and excellent flavor, are there any significant reasons to choose *Kele pig* as a model?

*Kele pigs* is a local characteristic pig species in the Wumeng Karst mountain area of the Yun-Guizhou Plateau, and belongs to a group of *Wujin pigs*. It is mainly produced in the northwest region of Guizhou. It is one of the local excellent germplasm resources in Guizhou, and has been included in the Annals of Chinese Livestock and Poultry Genetic Resources · Annals of Pigs, which is protected as a precious local breed resource in China. It has many advantages, such as high cold tolerance, strong stress resistance, rough feeding resistance and fine meat quality, but the low reproductive performance of *Kele pig* is one of the factors restricting the development and utilization of *Kele pig*. Therefore, this study aims to explore the genes related to collagen traits of *Kele pig* in addition to reproductive performance, so as to help the development and utilization of *Kele pig*.

4. The conclusion is not impressive in its current form. Conclusion section of the manuscript requires further development to strengthen its impact and clarity.

The conclusion of the manuscript is revised as follows, and has been revised in the revised manuscript and the manuscript revised.

Conclusion: Understanding the genes related to collagen-protein traits in *Kele pigs* may help us to further explore the regulatory factors of collagen-protein traits in *Kele pigs*. In this study, we reported the whole genome resequencing of five *Kele pigs*. Through SNPs and INDELs detection and annotation data analysis, genes related to collagen-protein traits of Guizhou *Kele pigs* were excavated. Including 307 candidate genes such as *COL9A1*, *COL6A5*, *COL4A4*, *COL4A3*, *EP300*, *SOS2*, *EPO*, etc., these genes may be potential targets for domestication, breeding, and selection in the past and future. In addition, through RT-qPCR analysis, we determined the expression levels of four candidate genes, *COL9A1*, *COL6A5*, *COL4A4* and *COL4A3*, in different tissues of *Kele pigs*, among which two genes, *COL9A1* and *COL6A5*, were significantly expressed in the skin tissues of *Kele pigs*. Both *COL4A4* and *COL4A3* genes were significantly expressed in the kidney tissue of *Kele pigs*. Our results are conducive to further research on collagen-protein trait regulation and development and utilization of Collagen-pig in the future. Our results are conducive to further research on the regulation of collagenous protein traits in *Kele pigs*.

According to the main and minor questions mentioned by reviewer 2, we have answered them as follows:

1. I did not find it relevant to utilize SNP/INDEL extraction from whole genome resequencing data to reveal the collagen related genes in *Kele pigs* from different tissues as it should have done using RNA-seq data as you are validating the change in level of expression using RT-PCR. Objective and conclusion of your study is failed to explain utilization of SNP/INDEL from whole genome resequencing data in place of RNA-seq data, which is the standard approach to access the expression of genes. Relevant explanation of it should be added and how its better to use SNP/INDEL extraction from whole genome resequencing data.

We greatly appreciate your professional evaluation of our articles. As you may be concerned, we have made changes to the previous manuscript and added some experiments to improve the quality of the manuscript. Based on the SNP mutation sites selected by whole genome resequencing, we randomly selected 14 SNP mutation sites for sanger sequencing verification, and the results showed that the selected mutation sites were consistent with the mutation sites previously screened, that is, G. 50509272G > C, G. 50509314G > T, G. 50509226C > T, G. 2117893T > C, G. 2117929T > G, G. 2118081C > T, G. 128531420C > G, G. 128531424C > T, G. 1285 31440G > A, G.128531456T > G, G.84423643G > A, G.84423793C > T, G.84423810g > A, G.84423811C > A, the 14 SNPs sites was the same as that of the SNP mutation sites screened by whole genome resequencing. The specific results are as follows:

**Fig 5 Peak map of SNPs locus with variation in Sanger sequencing of *COL9A1* gene**

**Fig 6 Peak map of SNPs locus with variation in Sanger sequencing of *COL6A5* gene**

**Fig 7 Peak map of SNPs locus with variation in Sanger sequencing of *COL4A4* gene**

**Fig 8 Peak map of SNPs locus with variation in Sanger sequencing of *COL4A3* gene**

2. Title, abstract and headings should be improved.

abstract has been revised, and the revised draft is as follows:

**Abstract:**【Objective】To verify the accuracy of collagen-specific SNP mutation loci of *Kele pigs* selected by whole genome resequencing, and to excavate collagen-related genes of *Kele pigs*, so as to lay a foundation for further molecular selection.【Methods】We randomly selected 14 Kele porcine specific SNPs sites from the whole genome retesting for sanger sequencing verification, and screened candidate genes related to collagen-protein traits of Kele porcine based on whole genome resequencing for gene annotation. Through KEGG and GO enrichment analysis of differential genes, we selected four genes that may affect collagen protein traits of collagen pigs, namely *COL9A1*, *COL6A5*, *COL4A3* and *COL4A4*. The expression levels of related genes were verified by RT-qPCR.【Results】Our sequencing results found that 241.04G of clean data, Q30 reached 93.96% and the average coverage depth was 9.04×, After data analysis, the SNP annotation of *Kele pigs* identified 4,570 high-impact mutation sites that could result in protein loss, with SNPs primarily distributed in the intronic and exonic regions. There were 132,256 middle-impact mutation sites and 318,150 low-impact mutation sites that could potentially impact protein properties. Additionally, The INDEL annotation results revealed a total of 17,806 high-impact mutation sites that could potentially result in the loss of protein function. There were 4740 medium-impact mutation sites that have the potential to affect protein properties, as well as 19,298 low-impact mutation sites. Furthermore, there were a staggering 14,197,763 modified-impact mutation sites identified in the analysis. 14 validated sites were randomly selected, That is, G. 50509272G > C, G. 50509314G > T, G. 50509226C > T, G. 2117893T > C, G. 2117929T > G, G. 2118081C > T, G. 128531420C > G, G. 128531424C > T, G. 1285 31440G > A, G.128531456T > G, G.84423643G > A, G.84423793C > T, G.84423810g > A, G.84423811C > A, the 14 SNPs sites was the same as that of the SNP mutation sites screened by whole genome resequencing. The results of real-time fluorescent quantitative PCR indicated that the expression levels of *COL4A4* and *COL4A3* in kidney tissue were found to be higher than those in other tissues. Additionally, we found that the expression levels of *COL9A1* and *COL6A5* in skin tissue were also higher compared to those in other tissues.【Conclusion】In this study, whole genome resequencing and data analysis were carried out on *Kele pigs* in Guizhou province. The specificity of 14 sites was consistent with the results of previous whole genome resequencing analysis, indicating that the specific SNP molecular marker information obtained by whole genome resequencing can be used as a basis for analyzing collagen-protein traits of *Kele pigs*. A total of 307 genes related to collagenic protein traits were excavated, including *COL9A1*, *COL6A5*, EP300, SOS2 and EPO, among which *COL9A1* and *COL6A5* genes were significantly expressed in the skin tissue of *Kele pigs*. Both *COL4A4* and *COL4A3* genes were significantly expressed in the kidney tissue of *Kele pigs*. Our results are conducive to further research on collagen trait regulation of *Kele pigs* and development and utilization of *Kele pigs* in the future.

3. There is no methodology provided for genomic localization (e.g. Exon etc.) of SNP/INDELs. Further, no methodology is provided to get impact of SNP/INDELs and for gene annotation of the SNP/INDELs as directly mentioned the KEGG analysis which is only possible after extraction of candidate genes of SNP/INDELs.

Methodology provided for genomic localization of SNP/INDELs has been supplemented and has been supplemented in the revised and manuscript as follows:

The variations were annotated using snpeff v5.0e software, and all SNPs were categorized into exons, introns, gene upstream regions, splicing sites, and other genomic regions based on their positions. SNPs classified into exon regions are further categorized as either synonymous variants or non-synonymous variants. The annotation process for INDELs is similar to that of SNPs, with the distinction between insertion and deletion regions based on their location in the genome. The body parameter is download-c./snpEff.config -v ebola_zaire Sscrofa11.1.99, -c./ snpEff.config-ud 2000-csvStats-htmlStats ". The annotation operation of INDEL is the same as that of SNPS, which are divided into insertion or deletion regions according to their location on the genome.

KEGG utilizes the R package clusterProfiler v4.0 to directly access the KEGG database official website (https://www.kegg.jp/) for KEGG analysis, and employs the built-in function of the R package dotplot for visual analysis. For GO analysis, it utilizes the R package org.Ss.Eg.Db v3.16.0 (http://bioconductor.org/packages/release/BiocViews.html#_OrgDb) and conducts visual analysis using the built-in function of clusterProfiler.

4. No detail of submission of raw sequencing data to NCBI.

We have uploaded the raw data to the ncbi website with the following details. (BioProject: PRJNA1140909)

Based on the main and secondary questions raised by reviewer 3, we have answered them as follows:

1. Line 111 *E.coli* should be in italics

Thank you for your careful inspection and correction, we are sorry for our carelessness. According to your comments, we have revised the manuscript as follows:

Then, the purified DNA fragments are inserted into the vector, and the library is transformed into *E. coli* by electrotransformation.

2. Line 195, 196, and 197 …what is G ?

Our study was rigorously filtered through sequencing data to produce high-quality clean data. Statistics of output data include sequencing data output, sequencing error rate, Q20 content, Q30 content, GC content, etc. G is a unit of data volume.

3. Linne 210 what is 9.04×

9.04× is the average sequencing depth of Kler pig samples in this study. Sequencing depth refers to the ratio of the total number of bases obtained by sequencing to the genome size, which is an important indicator for evaluating sequencing volume. This ratio can help evaluate the coverage and uniformity of sequencing data, thus affecting the accuracy and reliability of subsequent bioinformatics analysis.

4. Line 2-4 is not clearly specifying the objective of the study

We have made changes to the Objectivet section, as follows：

【Objective】To verify the accuracy of collagen-specific SNP mutation loci of *Kele pigs* selected by whole genome resequencing, and to excavate collagen-related genes of *Kele pigs*, so as to lay a foundation for further molecular selection.

5. Line 5-7 should be merged

We have modified this part as follows：

【Methods】We randomly selected 14 Kele porcine specific SNPs sites from the whole genome retesting for sanger sequencing verification, and screened candidate genes related to collagen-protein traits of Kele porcine based on whole genome resequencing for gene annotation. Through KEGG and GO enrichment analysis of differential genes, we selected four genes that may affect collagen protein traits of collagen pigs, namely *COL9A1*, *COL6A5*, *COL4A3* and *COL4A4*. The expression levels of related genes were verified by RT-qPCR.

6. Figure 5 labelling on X axis should be done in english

Sincerely thank you for your valuable comments, we have revised Fig 5 and corrected it in the revised manuscript and manuscript. (Fig 5 is changed to Fig 9 due to hand modification)

**Figure 9 Expression levels of *COL9A1*, *COL6A5*, *COL4A4* and *COL4A3* genes in different tissues of *Kele pigs***

7. Line 183 mentioned Figure 1 does not correlate with Fig 1 mentioned

Fig 1 illustrates that the extracted genomic DNA bands of *Kele pig* are singular and clear, with a high concentration.

**Fig 1 DNA quality test results of *Kele pig***

8. Line 185 past tense should be used

We have changed this part into the past tense.

Additionally, the DNA from all 5 samples meet the requirements for sequencing in the database.

9. Line 190 : Title doesn’t seem to be appropriate

We have revised the title to “Sequencing results”

10. Define G (Filter number) in line 201

We have added a definition for "G" as a "G" Data unit.

11. Line 213 and 214 should be explained more

PF data: During sequencing, llumina's built-in software determines whether to retain or discard the read based on the quality of the first 25 bases of each sequenced fragment (typically 100 bases long). If the quality control standard is not met, all the bases of the read are discarded; The data that meets the criteria and is retained is called PF data. data with quality score >=30 in PF data is called Q30 data. Q30 data: lllumina's built-in software judges the reliability of base identification results according to a uniform set of criteria, giving each base a quality score (QV). Q30 means that the reliability of the base is 99.9%, and Q30 data usually accounts for about 80% of PF data. According to the MiSeq standard parameter index of illumina, a high-throughput gene sequencing system in the United States, the sequencing quality standard is Q30, and more than 75% of the base proportion reaches Q30. Our research results show that Q30 of the filtered samples is higher than 90%, so it meets the quality control qualified data standard, and the sequencing data can be used for further analysis.

12. Line 216 to Line 222, including Table 4 should mention SNP’s and Indel with respect to each of the genome samples (Kel1, kel2, Kel3, kel4 and kel5)

In the manuscript, we have collected the overall data of five pigs, so that we can analyze the results more intuitively. For the individual data of kel1, kel2, kel3, kel4 and kel5, we have uploaded the original data to the NCBI official website for query.( BioProject: PRJNA1140909)

Based on the main and secondary questions raised by reviewer 5, we have answered them as follows:

1. The identification of high, medium, and low-impact mutation sites is thorough, with exact numbers provided. However, an explanation of how "impact" is defined and categorized would be beneficial for clarity.

We added how to get a high middle low impact method, as follows and has been supplemented in the revised and manuscript as follows:

The variations were annotated using snpeff v5.0e software, and all SNPs were categorized into exons, introns, gene upstream regions, splicing sites, and other genomic regions based on their positions. SNPs classified into exon regions are further categorized as either synonymous variants or non-synonymous variants. The annotation process for INDELs is similar to that of SNPs, with the distinction between insertion and deletion regions based on their location in the genome. The body parameter is download-c./snpEff.config -v ebola_zaire Sscrofa11.1.99, -c./ snpEff.config-ud 2000-csvStats-htmlStats ". The annotation operation of INDEL is the same as that of SNPS, which are divided into insertion or deletion regions according to their location on the genome.

The section on how to define influence is mentioned in the discussion section of the manuscript, as follows:

High-impact mutations can lead to the loss of protein function, while medium-impact mutations may impact the performance of the protein. Low-impact mutations are unlikely to affect protein function, and modified variants have no direct effect on gene and protein function.

2. The choice of tissues (kidney and skin) and the rationale behind selecting these tissues for expression analysis should be explained in more detail.

Collagen is a functional protein widely found in connective tissues such as bone, cartilage and skin of animals, and is an important component in maintaining the structure and function of tissues and organs such as skin, cartilage, tendons, ligaments and internal organs. China is a big country in livestock production and consumption, but most of the by-products of skin, bone and internal organs generated by slaughter are discarded, which are important sources of collagen. High-quality collagen and its functional polypeptide products produced abroad, such as medical devices and cosmetics and other high-value products are sold to China, while a large number of domestic animal collagen raw materials can only become low-value products or waste due to the incomplete deep processing industry chain, wasting a large amount of collagen raw material resources, and paying for premium products not made in China. Therefore, this study took *Kele pig* as an example to explore the expression levels of collagen-related genes in different tissues of heart, liver, spleen, lung, kidney, skin and longissimus dorsi muscle, laying a foundation for later exploration.

3. The manuscript reports extensive mutation data, including the identification of 4,570 high-impact SNPs, 132,256 medium-impact SNPs, and 318,150 low-impact SNPs. While these numbers are informative, it would be beneficial to include statistical measures such as mutation rates per base pair or per gene to provide a sense of scale and relevance.

As you may be concerned, we have made changes to the previous manuscript and added some experiments to improve the quality of the manuscript. Based on the SNP mutation sites selected by whole genome resequencing, we randomly selected 14 SNP mutation sites for sanger sequencing verification, and the results showed that the selected mutation sites were consistent with the mutation sites previously screened, that is, G. 50509272G > C, G. 50509314G > T, G. 50509226C > T, G. 2117893T > C, G. 2117929T > G, G. 2118081C > T, G. 128531420C > G, G. 128531424C > T, G. 1285 31440G > A, G.128531456T > G, G.84423643G > A, G.84423793C > T, G.84423810g > A, G.84423811C > A, and the specificity of the 14 SNPs sites was the same as that of the SNP mutation sites screened by whole genome resequencing.

4. For the INDEL annotation results, similar statistical measures would enhance the understanding of their significance. Including a comparison of observed mutation frequencies against expected frequencies could provide insights into whether certain types of mutations are overrepresented.
5. In the RT-qPCR analysis, it is important to report the statistical methods used to assess gene expression differences. Information on replicates, the statistical tests used (e.g., ANOVA, t-tests), and p-values or confidence intervals for the expression levels would strengthen the validity of the findings. Additionally, including measures of variance (such as standard error or standard deviation) for the expression levels would provide a clearer picture of the data's reliability.It is recommended to perform multiple testing corrections (e.g., Bonferroni or FDR) for the differential expression analysis to account for the possibility of Type I errors due to multiple comparisons.

Thank you very much for your correction. In the RT-qPCR experiment part of our study, we carried out six repeated experiments, and each repeated experiment carried out three individual repetitions, and the results were all consistent before we collected the results for statistics. In future experiments, we will correct them to make the experimental results more convincing.

6. For the KEGG and GO enrichment analyses, details on the statistical thresholds for significance (e.g., p-value cutoff, false discovery rate) should be provided. Including the enrichment scores and the number of genes in each pathway/category would also enhance the interpretability of the results.

Each enriched pathway and gene are quite numerous. The purpose of our study is to explore collagen-related genes in Kole pig, so we only select the genes that may be related to collagen for research as follows: (We can provide the overall data if necessary)

| **Serial number** | **KEGG pathway** | **Gene** |  |
| --- | --- | --- | --- |
| 1 | PI3K-Akt signaling pathway | *EGF*,*SPP1*,*FLT3*,*COL4A2*,*MAPK1*, *COL4A4*,*COL4A3*,*COL9A1*,*COL6A5* |  |
| 2 | Jak-STAT signaling pathway | *AKT2*,*CREBBP*,*EP300*,*OSMR*,*PIK3R1*, *PDGFRA*,*PDGFRB*,*PRL*,*PRLR* |  |
|  |  |  |  |
| 3 | MAPK signaling pathway | *AKT2*,*CD14*,*EPHA2*,*FOS*,*KITLG*, *KIT*,*MAPKAPK3*,*MKNK2*,*MECOM* |  |
| 4 | Wnt signaling pathway | *EP300*,*PPARD*,*DAAM2*,*NFATC4*,*DVL1*, *ROR1*,*PLCB2*,*INVS*,*RSPO2* |  |
| 5 | TGF-beta signaling pathway | *FBN1*,*THSD4*,*BMP4*,*TGFBR1*,*RBL1*, *CREBBP*,*MAPK1*,*TGFB2*,*RPS6KB2* |  |
| 6 | NF-kappa B signaling pathway | *TAB1*,*LTBR*,*IRAK4,RIPK1*,*PLCG2*, *LCK*,*TNFAIP3*,*TNFRSF11A*,*TLR4* |  |

7. The conclusion would benefit from a discussion on the broader implications of these findings for the breeding, health, and management of Kele pigs, as well as any potential applications in other pig breeds or species. To improve the manuscript, consider expanding the discussion on how these genetic discoveries can be applied practically, both within the context of Kele pigs and in a broader agricultural or biomedical context.

We have revised the discussion part, added the experimental part and explained it.

**Discussion**

Based on the sequencing data and reference genome, a total of 50,040,8.74 million SNPs loci and 14,216,261 INDELs loci were identified. The variations were predominantly found to be distributed in intergenic and intronic regions in this study. Intergenic variation may impact gene expression, RNA post-transcriptional modification, transcription factor binding, and splicing. Variation within introns may influence the occurrence of splicing events, thereby impacting the amino acid sequence that encodes for proteins and ultimately the structure and function of proteins. Ryu et al. ^[17]^ identified stroke-related SNPs in the gene spacer region between *FOXF2* and *FOXQ1* in zebrafish. These SNPs were found to regulate the enhancer activity and expression of the vascular stability regulator *FOXF2*, thus playing a role in regulating stroke risk in both human cells and zebrafish. High-impact mutations can lead to the loss of protein function, while medium-impact mutations may impact the performance of the protein. Low-impact mutations are unlikely to affect protein function, and modified variants have no direct effect on gene and protein function. The number of high-impact mutation sites in *Kele pigs* was 17,806, which can be highly disruptive to gene or protein function. In the context of functional mutation types of SNPs, non-synonymous mutations have the potential to alter the amino acid sequence, thereby impacting the structure and function of the protein. According to the findings of this study, a total of 134,584 non-synonymous mutations were identified in *Kele pigs*. In addition, base translocations can also result in significant changes in gene function, where a single base is substituted by another in a DNA sequence mutation. If both of the substituted bases are purines or pyrimidines, it is referred to as Transition (Ts); if one of the two replaced bases is a purine and the other is a pyrimidine, it is known as Transversion (Tv). This study identified 25,399,662 transversion points in *Kele pigs*. Liu et al. ^[18]^ demonstrated that a single non-synonymous mutation in the E-protein coding sequence 9 of the Zika virus can significantly increase neurovirulence in vivo. Similarly, Matsumoto et al. ^[19]^ found that non-synonymous mutations in the bovine *SPP1* gene may impact muscle development, leading to an increase in carcass weight of C/T animals. Therefore, non-synonymous mutations can alter protein structure and function for both beneficial and detrimental effects. Further investigation of the high-impact mutation sites, non-synonymous mutation sites, and translocation sites identified in this study can provide a better understanding of gene function and regulatory mechanisms in *Kele pigs*. INDEL refers to the insertion or deletion of a base in the DNA sequence. These variations can impact the function, structure, and genetic characteristics of the genome, playing a crucial role in genetic breeding exploration. Niu et al. ^[20]^ discovered that inserting 12 bases into the 3'UTR region of the *CISH* gene in Landwhite pigs could affect the susceptibility of landwhite piglets to diarrhea. Mi et al. ^[21]^ also found that three INDEL variation sites (L-13, L-16, and L-19) on the gene of cilia and flagella-related protein *CFAP43* were significantly correlated with growth traits such as chest depth in goats. In this study, a total of 7,041,172 insertion-deletion markers and 17,806 high-impact mutation sites were identified in *Kele pigs*. The discovery of these INDEL variation sites holds significant importance for comprehending the structure and function of the *Kele pig* genome. Furthermore, it provides valuable information for gene editing and genetic modification. We used sanger sequencing technology to verify the specific loci of *Kele pigs* previously screened. This verification method has also been reported in other studies. For example, Zhu Tao et al. ^[22]^ selected a total of 56 ducks from 7 populations, namely Cherry Valley Duck, Beijing duck, Maple Leaf duck, Jinding duck, Shaoxing duck, Shanma duck and Gaoyou duck, and conducted whole genome resequencing. 686 SNPs and Indel existing in specific populations were screened, and 7 SNPs sites were randomly selected for verification. The results were consistent with expectations. The specific SNPS screened in the second generation sequencing could be used as molecular markers for the identification of duck breeds. Fan Huanhuan et al. ^[23]^ randomly selected 30 red deer specific SNPs sites for sanger sequencing based on genotyping sequencing technology, and the research results indicated that red deer specific SNPs screened by GBS sequencing could be used as molecular markers for identification. This study is based on whole genome sequencing. The pathways involved in the regulation of collagen were screened, including the PI3K-Akt signaling pathway, Jak-STAT signaling pathway, MAPK signaling pathway, Wnt signaling pathway, TGF-beta signaling pathway, NF-kappa B signaling pathway, etc. A total of 133 genes were identified in the PI3K-Akt signaling pathway to regulate collagen-related traits in *Kele pigs*. Whole gene resequencing and GO analysis were utilized to investigate the genes associated with collagen deposition in *Kele pigs*, resulting in the identification of a total of 307 major candidate genes. The results revealed that these related genes were significantly enriched in several signaling pathways, including the PI3K-Akt signaling pathway, Jak-STAT signaling pathway, MAPK signaling pathway, Wnt signaling pathway, TGF-beta signaling pathway, and NF-kappa B signaling pathway. The PI3K-Akt signaling pathway regulates a variety of cellular processes in response to extracellular signals, including metabolism, proliferation, cell survival, growth, and angiogenesis ^[24]^. The MAPK signaling pathway plays a critical role in cell proliferation, differentiation, apoptosis, and metabolism ^[25]^.Hu et al. ^[26]^ showed that LPS promoted collagen synthesis in lung fibroblasts by activating the PI3K-Akt-mTOR/PFKFB3 pathway and aerobic glycolysis. Therefore, from the PI3K-Akt signaling pathway, *COL9A1*, *COL6A5*, *COL4A4* and *COL4A3* genes related to collagen-deposition in *Kele pigs* were preliminatively screened. In recent years, there have been many studies on the correlation between *COL9A1* and Kashin-beck disease ^[27]^, congenital clubfoot ^[28]^ and tumors. Currently, *COL6A5* gene has been associated with lipid metabolism ^[29]^, proliferation and angiogenesis of colon cancer cells ^[30]^. The study of Miner et al. ^[31]^ showed that gene mutations encoded by *COL4A3* and *COL4A4* could lead to glomerular diseases in humans and mice.

According to literature, the protein content of pig skin is reported to be as high as 33%, with collagen accounting for 87.7% of this total ^[32]^. In summary, this study randomly selected 14 SNP-related variation sites through whole genome resequencing analysis and conducted sanger sequencing to verify that the results were in line with expectations. That is, G. 50509272G > C, G. 50509314G > T, G. 50509226C > T, G. 2117893T > C, G. 2117929T > G, G. 2118081C > T, G. 128531420C > G, G. 128531424C > T, G. 1285 31440G > A, G.128531456T > G, G.84423643G > A, G.84423793C > T, G.84423810G > A, G.84423811C > A These mutation sites can be used as a reference for marker assisted selection. We initially explored the genes closely related to collagen-protein traits of *Kele pigs*, and detected the expression of genes *COL9A1*, *COL6A5*, *COL4A4* and *COL4A3* regulating collagen-protein traits in the heart, liver, spleen, lung, kidney, longissimus dorsi muscle and skin tissues of *Kele pigs* by qRT-PCR method. The results showed that both *COL9A1* and *COL6A5* genes were significantly expressed in the skin tissue of *Kele pigs*, and both *COL4A4* and *COL4A3* genes were significantly expressed in the kidney tissue of *Kele pigs*. The study laid the groundwork for further investigation into the regulation mechanism of collagens by *COL9A1*, *COL6A5*, *COL4A4*, and *COL4A3* genes. Based on the expression patterns of these genes in the heart, liver, spleen, lung, kidney, skin, and longissimus dorsi muscle of *Kele pigs*, it was found that *COL9A1* and *COL6A5* genes were significantly expressed in the skin of *Kele pigs*. This suggests a need for deeper exploration into how these genes regulate collagenic protein traits in Collagenic pigs. The findings provide a scientific basis for future research on the regulatory mechanisms of collagenic proteins related to Collagenic pig genes.

8. Minor grammatical corrections are needed in several areas for better readability. For example, "clean data of 241.04G" should be revised to "241.04 Gb of clean data," and "identified 4570 high-impact mutation sites" should be "identified 4,570 high-impact mutation sites."

【Results】Our sequencing results found that clean data of 241.04G

After data analysis, the SNP annotation of Kele pigs identified 4,570 high-impact mutation sites that could result in protein loss,

9. Line 9: "sequencing results found" should be "sequencing results revealed."Line 13: "could result in protein loss" should be "could result in protein function loss." Line 21: "modified-impact mutation sites" should be clarified or rephrased, as "modified-impact" is not a standard term in genetic annotation.

【Results】Our sequencing results revealed that 241.04 G of clean data, Q30 reached 93.96% and the average coverage depth was 9.04×. After data analysis, the SNP annotation of Kele pigs identified 4,570 high-impact mutation sites that could result in protein function loss, with SNPs primarily distributed in the intronic and exonic regions. There were 132,256 middle-impact mutation sites and 318,150 low-impact mutation sites that could potentially impact protein properties. Additionally, The INDEL annotation results revealed a total of 17,806 high-impact mutation sites that could potentially result in the loss of protein function. There were 4740 medium-impact mutation sites that have the potential to affect protein properties, as well as 19,298 low-impact mutation sites. Furthermore, there were 14,197,763 mutation sites of modification influence degree in the analysis.

Based on the main and secondary questions raised by reviewer 5, we have answered them as follows:

We have uploaded the raw data to the ncbi website with the following details. (BioProject: PRJNA1140909)

1. line no 6-9, more seems as results rather than methodology.

Thanks for your correction, we have made changes to this part as follows：

【Methods】We randomly selected 14 Kele porcine specific SNPs sites from the whole genome retesting for sanger sequencing verification, and screened candidate genes related to collagen-protein traits of Kele porcine based on whole genome resequencing for gene annotation. Through KEGG and GO enrichment analysis of differential genes, we selected four genes that may affect collagen protein traits of collagen pigs, namely *COL9A1*, *COL6A5*, *COL4A3* and *COL4A4*. The expression levels of related genes were verified by RT-qPCR.

1. Whether line 18-21, "There were 4740 medium-impact mutation sites
   19 that have the potential to affect protein properties, as well as 19,298
   20 low-impact mutation sites. Furthermore, there were a staggering
   21 14,197,763 modified-impact mutation sites identified in the analysis.", is required in abstract?

High-impact mutations can lead to the loss of protein function, while medium-impact mutations may impact the performance of the protein. Low-impact mutations are unlikely to affect protein function, and modified variants have no direct effect on gene and protein function. So we think this part needs to be mentioned, but we have also made changes to our manuscript summary section based on your suggestions.

2. At line 1191-192, authors mentioned that "After rigorous filtering of the sequencing data, high-quality clean,192 data was obtained.", they should also mention about that how 'rigorous filtering' was carried out

How to obtain high-quality clean data through strict filtering is elaborated in the part "Variation detection and filtering based on whole genome resequencing" in this manuscript. The specific method is: Fsatp v0.19.10 software is used to filter the original sequencing data, and the data obtained after removing adaptor and low-quality sequences is clean data.

3. line 198-200 needs restructuring
   We have modified this part as follows:

The Q30 of the filtered samples were all higher than 90%, which reached the quality control qualified data standard, and the sequencing data could be used for the next analysis.

4. line 216, "a total of 50,040,8.74 million SNPs loci and" is not clear!, at other places also it is mentioned.

The variation regions obtained by our study on Kel1, Kel2, Kel3, Kel4 and Kel5 samples are: exon714,620, Gene interval12,037,063, intron34,308,963, shear1,257, upstream1,114,108, downstream1,193,411,3 'end 506,819, 5 'end 164,633, so a total of 50,040,8.74 million SNPs sites.

5. whether in Table 4, mentioning "*Kele pig*" is needed as all the samples are of that only and table heading also mention it, same in Table 5, Table 6

Table 4 shows the Results of DNA OD value of *Kele pig*. "*Kele pig*" is not mentioned in the table. We named the five samples of *Kele pig* as Kel1, Kel2, Kel3, Kel4 and Kel5.

Table 4 Results of DNA OD value of *Kele pig*

| **Serial number** | | **Sample name** | | **Concentration**  **(ug/ml)** | | **OD260/280** | **OD260/230** |
| --- | --- | --- | --- | --- | --- | --- | --- |
| 1 | Kel1 | | 55.6 | | 1.85 | | 1.14 |
| 2 | Kel2 | | 378 | | 1.91 | | 1.96 |
| 3 | Kel3 | | 442 | | 1.93 | | 1.86 |
| 4 | Kel4 | | 812 | | 1.89 | | 1.5 |
| 5 | Kel5 | | 580 | | 1.97 | | 1.94 |

6. Line 236, "SNPS"!!

Thank you for your careful inspection and correction, we are sorry for our carelessness. According to your comments, we have revised the manuscript as follows:

A total of 24,971,814 SNPs were annotated, with 246,426 being synonymous mutations, accounting for 64.67% of the functional mutations.

7. line 270-271, "KEGG enrichment analysis was conducted on genes within the
   271 selected regions.", here also provide details about selected regions and their utilities

The KEGG enrichment analysis method is elaborated in detail in the "KEGG and GO annotation and channel analysis in *Kele pigs*" section of this manuscript. The specific methods are as follows:

KEGG utilizes the R package clusterProfiler v4.0 to directly access the KEGG database official website (https://www.kegg.jp/) for KEGG analysis, and employs the built-in function of the R package dotplot for visual analysis. For GO analysis, it utilizes the R package org.Ss.Eg.Db v3.16.0 (http://bioconductor.org/packages/release/BiocViews.html#_OrgDb) and conducts visual analysis using the built-in function of clusterProfiler.

8. line 292-"Kore"!

Thank you for your careful inspection and correction, we are sorry for our carelessness. According to your comments, we have revised the manuscript as follows:

Additionally, the expression of genes *COL9A1* and *COL6A5* in the skin tissue of *Kele pigs* was also found to be higher than that in other tissues (Fig 9).
