# Supplementary material for: Whole-genome resequencing reveals collagen-related genes in Kele pigs
Source: PLoS One. 2024 Dec 31;19(12):e0311417. doi: 10.1371/journal.pone.0311417 (PMC11687657; doi:10.1371/journal.pone.0311417)
Supplement: Attachment — Submitted filename: comments2.docx [file pone.0311417.s005.docx]

1. Headings in Experimental samples and methods section such as “Notes on Kele Pig SNPs and INDELs” should be replace with “Assessment of effects of SNPs and INDELs along with extraction of their associated genes”; and

“KEGG and GO annotation and channel analysis in Kele pigs” should be replaced with GO and KEGG analyses of genes associated with SNPs and INDELs”.

2. Additionally the content of heading “KEGG and GO annotation and channel analysis in Kele pigs” should also be improved.
3. There is no need to write results in each sub-heading of Result section.
4. Headings such as SNPs comment result and INDELs Comment Result should be also be improved.
