# Supplementary material for: Whole-genome resequencing reveals collagen-related genes in Kele pigs
Source: PLoS One. 2024 Dec 31;19(12):e0311417. doi: 10.1371/journal.pone.0311417 (PMC11687657; doi:10.1371/journal.pone.0311417)
Supplement: Attachment — Submitted filename: Response to Reviewers.docx [file pone.0311417.s006.docx]

Many thanks to all reviewers for their valuable comments and professional opinions on this study and us again. We sincerely thank the editors and all reviewers for their feedback and we will use this detailed feedback to improve the quality of the manuscript.

Based on the main and secondary questions raised by reviewer 1, we have answered them as follows:

Thanks for your recognition. With your valuable comments, our manuscript has been greatly improved. Thank you again.

Based on the main and secondary questions raised by reviewer 2, we have answered them as follows:

1. Headings in Experimental samples and methods section such as “Notes on *Kele Pig* SNPs and INDELs” should be replace with “Assessment of effects of SNPs and INDELs along with extraction of their associated genes”; and“KEGG and GO annotation and channel analysis in *Kele pigs*” should be replaced with GO and KEGG analyses of genes associated with SNPs and INDELs”.

Thank you for your valuable comments, which have greatly improved our manuscript. According to your comments, we have revised the manuscript as follows:

**Assessment of effects of SNPs and INDELs along with extraction of their associated genes**

The variations were annotated using snpeff v5.0e software, and all SNPs were categorized into exons, introns, gene upstream regions, splicing sites, and other genomic regions based on their positions. SNPs classified into exon regions are further categorized as either synonymous variants or non-synonymous variants. The annotation process for INDELs is similar to that of SNPs, with the distinction between insertion and deletion regions based on their location in the genome. The body parameter is download-c./snpEff.config -v ebola_zaire Sscrofa11.1.99, -c./ snpEff.config-ud 2000-csvStats-htmlStats ". The annotation operation of INDEL is the same as that of SNPS, which are divided into insertion or deletion regions according to their location on the genome.

**GO and KEGG analyses of genes associated with SNPs and INDELs**

KEGG utilizes the R package clusterProfiler v4.0 to directly access the KEGG database official website (https://www.kegg.jp/) for KEGG analysis, and employs the built-in function of the R package dotplot for visual analysis. For GO analysis, it utilizes the R package org.Ss.Eg.Db v3.16.0 (http://bioconductor.org/packages/release/BiocViews.html#_OrgDb) and conducts visual analysis using the built-in function of clusterProfiler.

2. Additionally the content of heading “KEGG and GO annotation and channel analysis in *Kele pigs*” should also be improved.

Thanks for your comments, we have revised this part as follows：

KEGG uses the R package clusterProfiler v4.0 to call the official website of KEGG database (https: //www.kegg.jp/)API) directly for KEGG analysis, and uses the built-in function of the R package dotplot for visual analysis. GO via R package org.Ss.eg.dbv3.16.0(http://bioconductor.org/packages/release/BiocViews.html#___OrgDb) is analyzed, and the use clusterProfiler built-in function dotplot visualization analysis; Finally through the online website and kish became the father of letter cloud tools dior (https: //www.omicshare.com/tools/Home/Soft/cog) into the line of visualization.

3. There is no need to write results in each sub-heading of Result section.

According to your comments, we have made the following modifications:

- Change "Quality test results of genomic DNA of *Kele pig*" to "DNA quality testing of samples of *Kele pig*"
- Change "Results of comparison between filter number and reference genome" to "Compare with the reference genome"
- Change "SNPs comment result ” and “ INDELs comment result" to "SNPs annotation results of *Kele pigs* " and "INDELs annotation results of *Kele pigs* "
- Change " SNP verification results " to " Verification results of SNP sites"

4. Headings such as SNPs comment result and INDELs Comment Result should be also be improved.

We have changed "SNPs comment result" and "INDELs comment result" to "SNPs annotation results of *Kele pigs*" and "INDELs annotation results of *Kele pigs* ”

5. Still, there are grammatical mistakes.

We are very sorry about this. Thanks for your suggestion. We will try our best to polish the language in the revised manuscript.

6. Abstract should be improved.

We have further corrected the summary, and the final version is as follows:

**Abstract**

【Objective】To verify the accuracy of collagen-specific SNP mutation loci of *Kele pigs* selected by whole genome resequencing, and to excavate collagen-related genes of *Kele pigs*, so as to lay a foundation for further molecular selection.【Methods】Based on whole genome resequencing, candidate genes related to collagen trait of *Kele pig* were screened for gene annotation. Through KEGG and GO enrichment analysis of differential genes, we selected four genes that may affect collagen trait of collagen pig, namely *COL9A1*, *COL6A5*, *COL4A3* and *COL4A4*. Then 14 specific SNP sites were randomly selected from the four genes for sanger sequencing verification, and finally RT-qPCR was used to verify the expression levels of related genes in different tissues of *Kele pigs*.【Results】Our sequencing results revealed that 241.04 G of clean data, Q30 reached 93.96% and the average coverage depth was 9.04×. After data analysis, the SNP annotation of *Kele pigs* identified 4,570 high-impact mutation sites that could result in protein function loss, with SNPs primarily distributed in the intronic and exonic regions. There were 132,256 middle-impact mutation sites and 318,150 low-impact mutation sites that could potentially impact protein properties. Additionally, The INDEL annotation results revealed a total of 17,806 high-impact mutation sites that could potentially result in the loss of protein function. There were 4740 medium-impact mutation sites that have the potential to affect protein properties, as well as 19,298 low-impact mutation sites. Furthermore, there were 14,197,763 mutation sites of modification influence degree in the analysis. In addition, through real-time fluorescence quantitative PCR results, we found that the expression levels of collagen-related genes *COL9A1* and *COL6A5* in skin tissues were higher than those in other tissues, and the expression levels of *COL4A4* and *COL4A3* in kidney tissues were higher than those in other tissues. The SNP site verification results showed that the 14 SNP mutation sites randomly selected by us were the same as the SNP mutation sites screened by whole genome resequencing.【Conclusion】A total of 307 genes related to collagen traits were excavated, including *COL9A1*, *COL6A5*, *EP300*, *SOS2* and *EPO*, etc. It was found that *COL9A1* and *COL6A5* genes were significantly expressed in the skin tissue of *Kele pigs*, and *COL4A4* and *COL4A3* genes were significantly expressed in the kidney tissue of *Kele pigs*. The mutations of 14 randomly selected loci in the four related genes were consistent with the results of previous whole genome resequencing analysis, indicating that the specific SNP molecular marker information obtained by whole genome resequencing can be used as the basis for analyzing collagen traits of *Kele pig*. Our results are conducive to further research on collagen trait regulation of *Kele pigs* and development and utilization of *Kele pigs* in the future.

7. In Introduction initial lines “Protein is the primary component of organismal structure and serves as the material basis for biological functions and metabolic activities. Its sequence contains a wealth of genetic information [1] .” could be removed as unnecessary.

We have deleted this sentence.

8. Heading “Validation of specific SNPs sites” could be replaced with “PCR based validation of 14 SNPs and their associated genes”.

We have changed " Validation of specific SNPs sites " to " PCR based validation of 14 SNPs and their associated genes "

9. Heading “Real-time fluorescent quantitative PCR” could be replace with “RT- fluorescent qPCR based validation of role of SNP associated genes in collagen expression”.

We have replaced "Real-time fluorescent quantitative PCR" with "RT-fluorescent qPCR based validation of role of SNP associated genes. in collagen expression"

10. Headings in Result section should be improved.

We have improved the title of some of the results, as follows:

- Change "Quality test results of genomic DNA of Kele pig" to "DNA quality testing of samples of Kele pig"
- Change "Results of comparison between filter number and reference genome" to "Compare with the reference genome"
- Change "SNPs comment result ” and “ INDELs comment result" to "SNPs annotation results of *Kele pigs* " and "INDELs annotation results of *Kele pigs* "
- Change " SNP verification results " to " Verification results of SNP sites"

Based on the main and secondary questions raised by reviewer 5, we have answered them as follows:

Reviewer #5: Manuscript needs a thorough modification as the work done is very interesting and of interest of wider community. Though the results are not properly presented and also not fully interpreted.

Also, I was not able to identify any information about the availability of raw or processed data generated in this manuscript.

We are very grateful to the experts for their careful review and detailed revision suggestions, which greatly improved our manuscript. We have uploaded our original data to ncbi for download and inspection. Here are the details of our raw data on the ncbi website.

(BioProject:PRJNA1140909)

1. line 177 : In order to verify the specific SNP sites of red deer ??

We feel sorry for our carelessness. In our resubmitted manuscript, the typo is revised. Thanks for your correction.

In order to verify the specific SNP sites of *Kele pigs*, 16 sites were randomly selected from the previously screened specific SNPs sites of Kele pig (Table 1) for verification.

2. line 181-182: According to the sequences of 100 bp before and after SNP sites were selected, according to what ??

Thank you for your valuable comments. After reviewing the literature, we found that some researchers^[1]^ chose to design a pair of PCR primers on both sides of the SNP in the sequencing verification experiment for the experiment, and this experiment used this method for reference. After the experiment, we found that this method could get the same result as our previous whole genome sequencing, so we used this method.

[1] Wu Yi-Jing, ZHANG Huiyi, Miao Chang-Chang, et al. Development and application of AS-PCR and D-CAPS markers for New Hupomelo variety 01-7 [J]. Journal of fruit trees, 2024, 9 (7) : 1310-1321. The DOI: 10.13925 / j.carol carroll nki GSXB. 20240146.

3. line 259-260: Additionally, the average sequencing depth of Kele pig samples was determined to be 9.04×.Initially, in materials and methods, it was mentioned as 10 X!!

We are very sorry for the misunderstanding caused by our expression. The sequencing depth of the method part is 10x, which is our expected sequencing depth. After the sequencing was completed, we analyzed and calculated the obtained data and found that the final sequencing depth was 9.04x, so the result part and the method part were not consistent.

4. line 275 SNPs comment result, is it an appropriate heading ??

line 302: INDELs Comment Result , again !!!

We have changed "SNPs comment result" and "INDELs comment result" to "SNPs annotation results of *Kele pigs*" and "INDELs annotation results of *Kele pigs* ”

5. line 408-409: "We used sanger sequencing technology to verify the specific loci of Kele pigs previously screened.", at line 136, Illumina is mentioned while at line 191-192, nothing is mentioned about sequencing technology!!

We have supplemented this. Thank you!

Finally, the PCR products identified correctly were sent to Qingke Biotechnology Co., Ltd. for sanger sequencing.

Our manuscript mentions Illumina, which is the method we used for whole genome resequencing, and sanger sequencing is the method we used to validate the mutation sites.

6. line 485: In this study, we reported the whole genome resequencing of five Kele pigs. authors are mentioned about whole genome resequencing but not much matter associated with that is available in the MS.

We are sorry to have confused you. In the experimental part of whole genome resequencing, we have explained it in the methods section. As follows:

**Variation detection and filtering based on whole genome resequencing**

Wu Han gene read Technology Co., LTD was commissioned to complete the whole genome resequencing of Kele pig. The main sequencing platform was Illumina HiSeq 2500, the sequencing strategy was PE125, and the depth was 10 x. The Fsatp v0.19.10 software was used to filter the original sequencing data, remove adaptor and low-quality sequences, and compare the filtered data to the porcine reference genome Sscrofa11.1 using bwa v0.7.17-r1188 software. The sequenced data was then de-duplicated to obtain a bam file for mutation detection. The read was compared to the pig reference genome (Sscrofa11.1) downloaded from the NCBI database using samtools vl.16.1 and sorted based on the length score of the comparison. Once sorting is complete, Picard MarkDuplicates v2.18.29-SNAPSHOT is utilized to eliminate duplicate comparisons. Use GATK v4.0 (https://gatk.broadinstitute.org/hc/en-us) HaplotypeCaller tools to respectively analyze the Kele pig bam files for mutation detection. The gvcf file for each pig was then merged into a single population gvcf file containing variation across the entire population.Filter SNP and INDEL using the VariantFiltration tool in GATK 4.0. The quality control filter parameters of SNPs and INDELs are QD < 2.0 || MQ < 40.0 || FS > 60.0 || SOR > 3.0 || MQRankSum < -12.5 || ReadPosRankSum < -8.0.
